# Co-infection With Chromosomally-Located *bla*_CTX-M-14_ and Plasmid-Encoding *bla*_CTX-M-15_ in Pathogenic *Escherichia coli* in the Republic of Korea

**DOI:** 10.3389/fmicb.2020.545591

**Published:** 2020-11-11

**Authors:** Jungsun Park, Eunkyung Shin, Ae Kyung Park, Soojin Kim, Hyun Ju Jeong, Jin Seok Kim, Young-Hee Jin, Nan Joo Park, Jeong-hoon Chun, Kyujam Hwang, Kwang Jun Lee, Junyoung Kim

**Affiliations:** ^1^Division of Bacterial Diseases, Center for Laboratory Control of Infectious Diseases, Korea Centers for Disease Control and Prevention, Chungju, South Korea; ^2^Infectious Diseases Team, Seoul Metropolitan Government Research Institute of Public Health and Environment, Seoul, South Korea; ^3^Microbiology Team, Gyeonggi-do Institute of Health and Environment, Suwon, South Korea; ^4^Division of Antimicrobial Resistance, National Institute of Health, Center for Infectious Diseases Research, Centers for Disease Control and Prevention, Chungcheongbuk-do, South Korea

**Keywords:** co-infection, CTX-M, pathogenic *Escherichia coli*, chromosomally-located *bla*_CTX-M-14_, plasmid-encoding *bla*_CTX-M-15_

## Abstract

The emergence of third-generation cephalosporin resistance in *Escherichia coli* is increasing at an alarming rate in many countries. Thus, the aim of this study was to analyze co-infecting *bla*_CTX-M_-producing pathogenic *E. coli* isolates linked to three school outbreaks. Among 66 *E. coli* isolates, 44 were identified as ETEC O25, an ETEC isolate serotype was O2, and the other 21 were confirmed as EAEC O44. Interestingly, six patients were co-infected with EAEC O44 and ETEC O25. For these isolates, molecular analysis [antibiotic susceptibility testing, identification of the β-lactamase gene, multilocus sequence typing (MLST), and pulsed-field gel electrophoresis (PFGE)] was performed for further characterization. In addition, the transmission capacity of *bla*_CTX-M_ genes was examined by conjugation experiments. Whole-genome sequencing (WGS) was performed on representative EAEC O44 and ETEC O25 isolates associated with co-infection and single-infection. All isolates were resistant to cefotaxime and ceftriaxone. All EAEC isolates carried the *bla*_CTX-M-14_ gene and all ETEC isolates the *bla*_CTX-M-15_ gene, as detected by multiplex PCR and sequencing analysis. Sequence type and PFGE results indicated three different patterns depending on the O serotype. WGS results of representative isolates revealed that the ETEC O25 strains harbored *bla*_CTX-M-15_ located on IncK plasmids associated with the Δ*bla*_TEM_-*bla*_CTX-M-15_-*orf477* transposon. The representative EAEC O44 isolates carried *bla*_CTX-M-14_ on the chromosome, which was surrounded by the IS*Ecp1*-*bla*_CTX-M-14_-*IS903* transposon. To the best of our knowledge, this is the first report of co-infection with chromosomally located *bla*_CTX-M-14_ and plasmid-encoding *bla*_CTX-M-15_ in pathogenic *E. coli*. Our findings indicate that resistance genes in clinical isolates can spread through concurrent combinations of chromosomes and plasmids.

## Introduction

Pathogenic *Escherichia coli* is a cause of gastroenteritis, including foodborne outbreaks, worldwide. Most *E. coli* infections are self-limiting, and sometimes require antimicrobial treatment. For treatment of *E. coli* infections, antibiotics such as third-generation cephalosporins and fluoroquinolones are prescribed (Kim et al., 2014b).

However, the emergence of antimicrobial resistance is increasing at an alarming rate in many countries. Furthermore, third-generation cephalosporin resistance, including resistance against cefotaxime and ceftriaxone, has been steadily reported in recent years. Additionally, many countries have rapidly experienced the dissemination of extended-spectrum-β-lactamase (ESBL)-producing *Enterobacteriaceae* isolates, particularly *E. coli* (Oteo et al., 2012).

Extended-spectrum-β-lactamase-producing *Enterobacteriaceae* appeared in the 1990s, and these bacteria have steadily become prevalent, being primarily associated with the *bla*_CTX-M-14_ and *bla*_CTX-M-15_ genes in the Republic of Korea (Naseer and Sundsfjord, 2011; Shin et al., 2011, 2012).

The first finding of CTX-M-14 and CTX-M-15 in clinical *E. coli* isolates was reported in 2001 and 2005 (Pai et al., 2001; Kim et al., 2005), respectively. Since then, a growing trend of CTX-M-producing clinical *E. coli* isolates has been observed (Kim et al., 2016, 2019).

The spread of CTX-M enzyme-coding capacity occurs due to the mobilizing ability of their insertion sequences and integrons (Oteo et al., 2010). Moreover, a majority of CTX-M-producing isolates can transfer ESBL plasmids between bacteria of the same and/or different species *via* horizontal transmission (Baker et al., 2018). Moreover, a number of chromosomally located CTX-M genes in *E. coli* have been reported in several studies, indicating that the CTX-M genes can be transferred *via* transposons or insertion sequences into the chromosome (Hamamoto and Hirai, 2019).

In this study, we describe *bla*_CTX-M-14_‐ and *bla*_CTX-M-15_-producing pathogenic *E. coli* associated with three school outbreaks in distinct regions in the Republic of Korea. In particular, several patients were co-infected with CTX-M-14-producing EAEC and CTX-M-15-producing ETEC during this outbreak period. Thus, the aim of this study was to analyze co-infecting *bla*_CTX-M_-producing pathogenic *E. coli*, including their resistance genes, genetic environments, and plasmid profiles.

## Materials and Methods

### Bacterial Isolates

In August 2017, local public laboratories reported an outbreak of acute diarrheal illness in three local high schools (school A in Siheung, school B in Gwangju, and school C in Seoul). These outbreaks affected 634 persons from the three different schools during the period 14 August–25 August. A total of 250 stool samples (216 from case patients and 34 from food handlers) and 140 environmental samples, including 92 preserved food products consumed during the outbreak period, 22 cooking utensils, and 16 drinking water samples, were collected and tested for bacteriological and virological assessments (Shin et al., 2015). A total of 66 stool samples were positive for *E. coli*, whereas the environmental samples and stool samples from staff of the three schools were negative for any pathogenic bacteria or virus.

The isolated *E. coli* strains were pathotyped by an 8-plex real-time PCR kit (Kogene Biotech, Seoul, South Korea) to detect specific virulence genes, such as VT1 and VT2 for enterohemorrhagic *E. coli* (EHEC), LT, and sequence type (ST) for enterotoxigenic *E. coli* (ETEC), *eaeA* and *bfpA* for enteropathogenic *E. coli* (EPEC), *ipaH* for enteroinvasive *E. coli* (EIEC), and *aggR* for enteroaggregative *E. coli* (EAEC), according to the manufacturer’s instructions (Shin et al., 2016). All isolates were serotyped by a slide agglutination test using *E. coli* antiserum for the O antigens (Denka Seiken, Tokyo, Japan).

### Antimicrobial Susceptibility Testing and Identification of the β-Lactamase Gene

Antimicrobial susceptibility testing of the 66 *E. coli* isolates was performed using the broth microdilution method with customized Sensititre KRCDC1F panels (TREK Diagnostic Systems, East Grinstead, United Kingdom) in accordance with the guidelines established by the Clinical and Laboratory Standards Institute (CLSI). The antimicrobial agents tested were ampicillin, azithromycin, amoxicillin/clavulanic acid, cefoxitin, ceftazidime, ceftriaxone, cefotaxime, imipenem, gentamicin, amikacin, streptomycin, tetracycline, nalidixic acid, ciprofloxacin, trimethoprim/sulfamethoxazole, and chloramphenicol.

In the case of cefotaxime‐ or ceftriaxone-resistant isolates, the presence of ESBL genes was confirmed by multiplex PCR for TEM, SHV, CMY, OXA, DHA, and CTX-M types, and CTX-M-type genes were characterized by sequencing analysis of the amplicons (Kim et al., 2009).

### Multilocus Sequence Typing

All isolates were assigned by multilocus sequence typing (MLST) as previously described (Wirth et al., 2006). Seven housekeeping genes (adkA, fumC, gyrB, icd, mdh, purA, and recA) were sequenced following the protocols specified on the *E. coli* MLST website.^1^ The primer sequences are available at http://enterobase.warwick.ac.uk/species/ecoli/download_7_gene. Seven different gene fragments of each isolate were assigned an allele number, and the sequence type (ST) was determined by each unique combination of seven allelic profiles.

### Pulsed-Field Gel Electrophoresis

All *E. coli* isolates were analyzed by pulsed-field gel electrophoresis (PFGE) after *Xba*I digestion, according to the PulseNet International protocol.^2^ Fragments of *Xba*I-digested DNA were separated using a CHEF-Mapper system (Bio-Rad, Hercules, CA, United States) at 6 V/cm, with a linear increase in switching times from 2.16 to 54.17 s over 18 h at 14°C. Genetic similarities between the PFGE patterns were calculated with BioNumerics v7.6 (Applied-Maths, Sint-Martens-Latem, Belgium) using the Dice coefficient with a 1.5% band tolerance and the unweighted-pair group method using arithmetic averages (UPGMA).

### Whole-Genome Sequencing

To determine genetic characteristics, representative isolates were selected according to their serotypes, antimicrobial resistance patterns, sequence types, PFGE results, and *bla*_CTX-M_ genes. Genomic DNA was isolated using a Blood and Tissue kit (Qiagen, Stockach, Germany) according to the manufacturer’s protocol. DNA purity was quantified using a NanoDrop 2000 spectrophotometer (Thermo Fisher, DE, United States) and a Qubit 4 fluorometer using a high-sensitivity kit (Invitrogen, CA, United States). Short-read sequencing libraries were prepared with an Illumina Nextera Flex library preparation kit (Fonteneau et al., 2017). Sequencing was performed using a MiSeq sequencer (Illumina, San Diego, CA, United States) to generate 250-bp paired-end reads according to the manufacturer’s instructions. A long-read MinION sequencing library was prepared by using the ligation sequencing kit (SQK-LSK109) according to the manufacturer’s protocol for genomic DNA. Sequencing was carried out using a version R9.4.1 flow cell (FLO-MIN 106D).

### Data Analysis and Molecular Characterization

The raw sequences generated by Illumina MiSeq were quality filtered using FastQC, with the average quality set at Q30. The contigs of genomic sequences were *de novo* assembled with a minimum contig size threshold of 200 bp using SPAdes assembler v3.9.0 (Abdalhamid et al., 2019). The raw data generated by the MinION instrument were processed, and base calling was performed using guppy software version 2.3.7; long-read assembly was performed using CLC genomics workbench 20.0.3. Subsequently, a hybrid *de novo* assembly of Illumina and Nanopore reads was performed using the long read support tool within the CLC genomic workbench 20.0.3. Genome annotations were performed using Rapid Annotation using Subsystem Technology (RAST). Assembled sequences were analyzed using bioinformatics web tools available from the Center for Genomic Epidemiology (CGE) website^3^ to detect resistance genes (ResFinder 3.2) and identify plasmid replicon types (PlasmidFinder 2.1). These whole-genome sequence data have been deposited in the National Center for Biotechnology Information (NCBI) under the Bio-Project PRJNA595397. The GenBank accession numbers are listed in Supplementary Table 1.

### Mating Experiments and Plasmid Analysis

Strains exhibiting cefotaxime resistance were examined by conjugation experiments using azide-resistant *E. coli* J53 as the recipient strain to confirm the transmission capacity of *bla*_CTX-M_. Transconjugants were selected on MacConkey agar plates (Difco, United States) supplemented with cefotaxime (1 μg/L) and sodium azide (200 μg/L), and putative transconjugants were confirmed by antimicrobial susceptibility tests. In addition, transconjugants were selected according to their incompatibility group determined by PCR-based replicon typing (PBRT) and their acquisition of the *bla*_CTX-M_ gene. Whole-genome DNA from transconjugants was digested with S1 endonuclease (Thermo Fisher Scientific, MA, United States) and incubated at 37°C for 30 min to estimate their plasmid sizes. DNA fragments were separated by PFGE through a CHEF-Mapper system for 14 h at 6 V/cm, with initial and final pulse times of 1 and 25 s.

## Results

### Bacterial Isolates

The RT-PCR results for 66 *E. coli* strains indicated that 45 carried the ST gene and were classified as ETEC (45, 68.2%); 21 strains carried the *aggR* gene and were confirmed as EAEC (21, 31.8%). A total of 44 ETEC strains were classified as O-type O25, and the serotype of one ETEC strain was O2; the other 21 strains were confirmed as O44. Additionally, we confirmed that six patients were co-infected with ETEC O25 and EAEC O44.

### Antimicrobial Susceptibility Testing and ESBL Gene Analysis

All of the *E. coli* isolates were resistant to cefotaxime and ceftriaxone. In addition, the EAEC O44 isolates were resistant to tetracycline, chloramphenicol, azithromycin, and trimethoprim/sulfamethoxazole. The ETEC O25 isolates were resistant to azithromycin, and ETEC O2 isolates were resistant to tetracycline, chloramphenicol, and trimethoprim/sulfamethoxazole. The co-infection isolates from the six patients displayed the same resistance patterns as the ETEC O25 and EAEC O44 isolates (Table 1). PCR analysis showed that all EAEC O44 isolates harbored *bla*_CTX-M-14_ and that both ETEC O25 and O2 isolates carried *bla*_CTX-M-15_.

**Table 1:** Antimicrobial susceptibility profiles of EAEC O44, ETEC O25, and ETEC O2, representative strains of three school outbreaks, and the transconjugant strains ETEC O25-TC and ETEC O2-TC.

Antimicrobial agents (S)	MIC, μg/L					
	EAEC O44 (*n* = 44)	ETEC O25 (*n* = 21)	ETEC O2 (*n* = 1)	*Escherichia coli* J53	ETEC O25-TC	ETEC O2-TC
Ampicillin	>64	>64	>64	>64	>64	>64
Azithromycin	8	4	4	4	4	4
Amoxicillin-clavulanic acid	8/4	4/2	4/2	4/2	4/2	4/2
Cefoxitin	<2	<2	<2	<2	<2	<2
Ceftazidime	<1	<1	<1	<1	<1	<1
Ceftriaxone	16	>32	16	<1	32	16
Cefotaxime	32	>32	16	<1	32	16
Imipenem	<1	<1	<1	<1	<1	<1
Gentamicin	<1	<1	<1	<1	<1	<1
Amikacin	<4	<4	<4	<4	<4	<4
Streptomycin	8	8	8	4	4	4
Tetracycline	128	<2	>128	<2	<2	<2
Nalidixic acid	<2	8	<2	<2	<2	<2
Ciprofloxacin	<1	0.5	<1	<1	<1	<1
Trimethoprim-sulfamethoxazole	>16/304	<2/1	>16/304	<2/1	<2/1	<2/1
Chloramphenicol	>32	8	>32	<4	<4	<4

### MLST and PFGE Analysis

**Figure 1:**
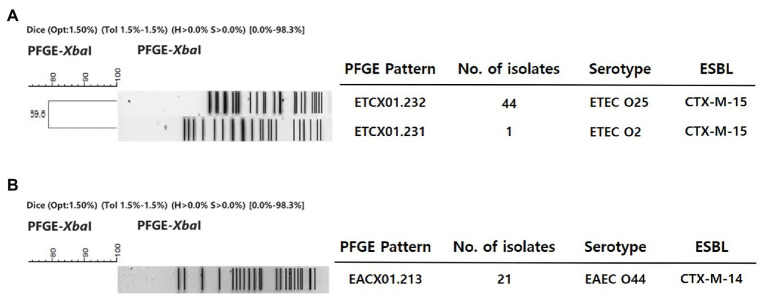
Dendrogram of the *Xba*I-pulsed-field gel electrophoresis (PFGE) patterns of enterotoxigenic *E. coli* (ETEC) and enteroaggregative *E. coli* (EAEC) strains isolated from the clinic. This dendrogram was constructed with BioNumerics v5.1 (Applied-Maths, Belgium) using the unweighted-pair group method with arithmetic means and a Dice coefficient (1.5% optimization and 1.5% position tolerance). **(A)** ETEC. **(B)** EAEC.

The identified MLSTs of the EAEC O44, ETEC O25, and O2 strains were ST414, ST1492, and ST95, respectively. All ETEC O25 isolates displayed an identical *Xba*I digestion pattern (ETCX01.232), except for a strain with a different serotype, which was an ETEC O2 isolate exhibiting an ETCX01.231 *Xba*I digestion pattern (Figure 1). All of the EAEC O44 isolates showed the same *Xba*I digestion pattern: EACX01.213 (Figure 1). These three patterns were identified as new patterns that do not match any known PFGE cluster in the PulseNet Korea database of *E. coli* strains.

### Genetic Profiles of *E. coli* Isolates

EAEC O44 and ETEC O25 co-infecting and single-infecting isolates were selected for further experiments and divided into two types according to serotypes, antimicrobial resistance patterns, sequence types, PFGE results, and *bla*_CTX-M_ genes. Twelve isolates were chosen for the comparison of the genetic characteristics of co-infecting and single-infecting isolates: six EAEC O44 isolates and six ETEC O25 isolates, including eight co-infecting isolates from four patients.

To obtain a highly accurate closed complete genome sequence of the six EAEC O44 and six ETEC O25 strains, hybrid *de novo* assembly was performed using Nanopore long reads and Illumina short reads. The selected representative EAEC O44 and ETEC O25 isolates showed an identical genetic characteristic. The representative EAEC O44 strains were found to contain a 5.3-Mb circularized chromosome and three plasmids ranging from 90,954 to 99,334 bp. The *bla*_CTX-M-14_ genes mediating resistance in representative EAEC O44 isolates are located on the chromosome, and additional resistance genes, including for tetracyclines [*tet(A)*], phenicols (*catA1*), macrolides [*mdf(A)*], trimethoprim (*dfrA5*), and sulfonamides (*sul1*), are also present on this chromosome. Three plasmids were confirmed to be IncFIC(FII) plasmids with no resistance genes (Table 2).

**Table 2:** Overview of antibiotic resistance genes, virulence genes and multilocus sequence typing (MLST) detected for representative EAEC O44 and ETEC O25 isolates associated with co-infection and single-infection.

Strain	Virulence gene	MLST	Contig	Size (bp)	Plasmid type	Antibiotic resistance genes
EAEC_O44	*aggR*	ST414	Chromosome	5,326,743	-	*bla*_CTX-M-14_, *tet(A)*, *catA1*, *mdf(A)*, *dfrA5*, *sul1*
			Plasmid pEAEC_O44_1	91,298	IncFIC(FII)	None
			Plasmid pEAEC_O44_2	99,335	IncFIC(FII)	None
			Plasmid pEAEC_O44_3	91,263	IncFIC(FII)	None
ETEC_O25	ST	ST1491	Chromosome	5,153,435	-	*mdh(A)*
			Plasmid pETEC_O25_1	98,083	IncK	*bla*_CTX-M-15_
			Plasmid pETEC_O25_2	104,278	IncFII/FIB	None

The selected ETEC O25 strains exhibit a circular chromosome and two plasmids of 98,076 and 104,278 bp. Each plasmid type was confirmed to be IncK (repZ) and IncF (repFIB/repFII) based on the plasmid replicons by PlasmidFinder. The IncK replicon plasmid of a selected ETEC O25 isolate carries a resistance gene for β-lactams, *bla*_CTX-M-15_, at positions 9,141–10,016 bp; additional resistance genes, such as *mdh(A)* for macrolides, were detected on the chromosome. The IncF replicon plasmid carries two replicons genes (repFIB and repFII) in a same plasmid and no antibiotic resistance genes (Table 2).

### Analysis of Regions Surrounding *bla*_CTX-M_

**Figure 2:**
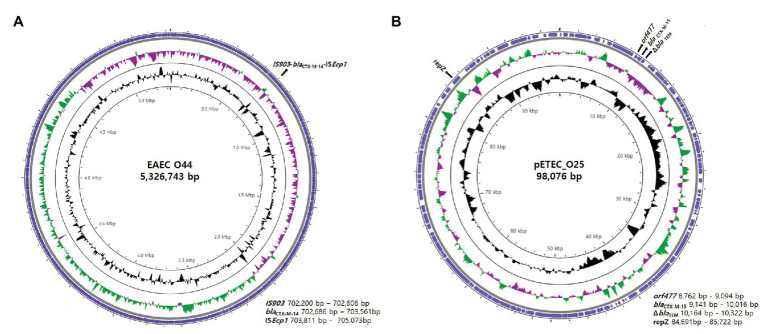
Genetic overview of EAEC O44 isolates carrying chromosomally located *bla*_CTX-M-14_ and ETEC O25 isolates harboring plasmid-encoded *bla*_CTX-M-15_. Circular genome map representation. The outermost ring represents the blast results for EAEC O44 and ETEC O25. G + C content, black peak; G + C positive skew, green peak; G + C negative skew, purple peak. **(A)** EAEC_O44: the EAEC O44 genome is 5,326,743 bp, and the IS*Ecp1*-*bla*_CTX-M-14_-*IS903* transposon (2,874 bp) is located between 702,200 and 705,073 bp on the chromosome. **(B)** pETEC_O25: pETEC_O25 is 98,076 bp in size, and the Δ*bla*_TEM_-*bla*_CTX-M-15_-*orf477* transposon (1,540 bp) was found on the IncK plasmid between positions 8,762 and 10,322 bp.

**Figure 3:**
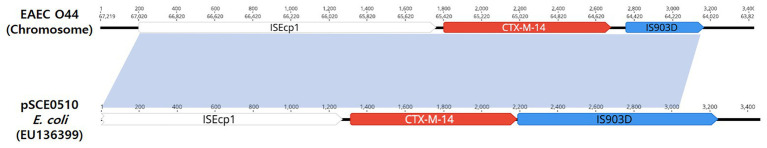
Schematic representation of the genetic environment surrounding the *bla*_CTX-M-14_ gene. The hatched box and the arrow indicate the genetic structure IS*Ecp1*-*bla*_CTX-M-14_-*IS903*.

Combining long-read and short-read sequencing results indicated that the representative EAEC O44 genome is 5,326,743 bp and that the IS*Ecp1*-*bla*_CTX-M-14_-*IS903* transposon is located between 702,200 and 705,073 bp on the chromosome (Figure 2). The EAEC O44 isolates carry the IS*Ecp1*-*bla*_CTX-M-14_-*IS903* transposon (2,874 bp), in which IS*Ecp1* is located upstream of the start codon of the *bla*_CTX-M-14_ gene and the *IS903* sequence in the downstream region (Figure 3).

The results for selected ETEC O25 isolates indicated the transposon region (Δ*bla*_TEM_-*bla*_CTX-M-15_-*orf477*) to be located on the IncK plasmid between positions 8,762 and 10,322 bp (Figure 2).

**Figure 4:**
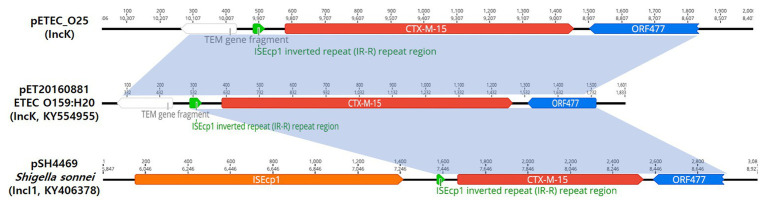
Schematic representation of the genetic environment surrounding the *bla*_CTX-M-15_ gene on three plasmids. The hatched box and the arrow indicate the inverted repeat (IR) of the IS*Ecp1* element and the transcriptional start site for the *bla*_CTX-M-15_ gene, respectively.

The representative isolates of *bla*_CTX-M-15_-producing ETEC O25 include the Δ*bla*_TEM_-*bla*_CTX-M-15_-*orf477* transposon (1,540 bp) as the genetic structure. Analysis of the region flanking *bla*_CTX-M-15_ revealed an *orf477* sequence upstream and downstream of the *bla*_CTX-M-15_ gene, and the spacer region between the inverted repeat (IR) sequences of IS*Ecp1* and a truncated *bla*_TEM_ gene was observed (Figure 4).

### Transmission of the *bla*_CTX-M_ Gene

Conjugation experiments were performed with all cefotaxime‐ and ceftriaxone-resistant isolates, which served as donors and *E. coli* J53 as the recipient. The results indicated that both ETEC O2 and ETEC O25 isolates were able to transfer resistance to recipient *E. coli* cells. According to the minimal inhibitory concentrations (MICs) of the antimicrobials, the ETEC transconjugants exhibited resistance to cefotaxime and ceftriaxone (Table 1). Additionally, PCR amplification and sequencing results confirmed that the *bla*_CTX-M-15_ gene was present in all of the ETEC transconjugants. Analysis of PBRT of all transconjugants demonstrated that the CTX-M-15-producing ETEC isolates carried two replicons of IncK and IncF (FII and FIB). These results supported by S1-PFGE analysis showed two plasmids ranging from 78 to 104 kb. Based on WGS analysis, two plasmids were present in the ETEC O25 strains, IncFII, FIB (104 kb), and IncK (98 kb), with *bla*_CTX-M-15_ located on the IncK plasmid. Conversely, the mating experiments with EAEC isolates failed to yield transconjugants.

## Discussion

According to surveillance for foodborne and waterborne infection outbreaks during the period of 2013–2017 in the Republic of Korea, an estimated 7,600 cases occurred annually, among which 8.7% were associated with multiple pathogen outbreaks involving co-infection (Korea Centers for Disease Control & Prevention, 2013, 2014, 2015, 2016, 2017). In addition, several cases of co-infection with dual pathogens have been steadily reported in other countries (Wensley and Coole, 2013; Ahmed et al., 2014).

In this study, we describe co-infection with chromosomally located *bla*_CTX-M-14_‐ and plasmid-encoding *bla*_CTX-M-15_-producing pathogenic *E. coli* associated with three outbreaks. These outbreaks occurred at three schools located in distinct regions in the Republic of Korea. Based on an epidemiological investigation, the main cause of this outbreak was linked to the consumption of imported food (kimchi) supplied by the same food company. This assumption was supported by an outcome questionnaire that indicated that food (kimchi) was significantly related to illness. The results of laboratory tests suggested that these strains might have been introduced into the common source of infection. As follow-up actions in response to the described outbreak, the local public health laboratories launched an additional investigation of the food company. Unfortunately, however, food items contaminated by pathogenic *E. coli* were not identified in this investigation.

Interestingly, these outbreaks have been associated with EAEC O44 and ETEC O25 as co-infecting and single-infecting isolates. These isolates were divided into two types based on O serotype, antimicrobial resistance patterns, MLST, *Xba*I digestion patterns, and *bla*_CTX-M_ genes. Additionally, by combining long-read and short-read sequencing results, we confirmed identical results for the selected representative co-infecting and single-infecting EAEC O44 and ETEC O25 isolates.

Each representative EAEC O44 and ETEC O25 strain was confirmed to produce the *bla*_CTX-M-14_ gene located on the chromosome and carry the *bla*_CTX-M-15_ gene on the IncK plasmid. Additionally, several patients were identified with a co-infection of both *bla*_CTX-M-14_-producing EAEC O44 and *bla*_CTX-M-15_-producing ETEC O25. The difference between the EAEC O44 and ETEC O25 strains was that the latter transmitted resistance *via* horizontal transmission between the chromosome and resident plasmids with a *K* replicon.

The prevalence of chromosomally located *bla*_CTX-M-14_ genes in clinical *E. coli* isolates from Europe and Asian countries has been observed in previous studies (Hawkey and Jones, 2009; Rodríguez et al., 2014; Hamamoto et al., 2016). Notably, chromosomal integration of *bla*_CTX-M-14_ genes in *E. coli* strains has been described in the Republic of Korea (Figure 3; Kim et al., 2011). These results support the hypothesis that the *bla*_CTX-M-14_ gene was chromosomally integrated into *E. coli* strains *via* transposable elements, such as IS*Ecp1*-like elements (Hawkey and Jones, 2009). However, the phenomenon of dissemination of the chromosomal location of the *bla*_CTX-M-14_ gene in *Enterobacteriaceae*, including *E. coli* isolates, remains unclear, as has been the case in previous studies (Rodríguez et al., 2014; Hamamoto et al., 2016).

In contrast, the representative isolates of *bla*_CTX-M-15_-possessing ETEC O25 were found to harbor IncK plasmids. The *bla*_CTX-M-15_ gene is located between 9,141 and 10,016 bp on the plasmid, with an *orf477* sequence downstream, but none of the transposable elements frequently associated with *bla*_CTX-M_ were observed in the upstream region. The overall genetic structure of the selected ETEC O25 isolates (*Δbla*_TEM_-*bla*_CTX-M-15_-*orf477*) has been described to be similar to that of pET20160881 (IncK, GenBank accession number KY554955; Figure 4) and pSH4469 (IncI1, GenBank accession number KY406378; Figure 4; Kim et al., 2014a, 2017). The plasmid backbone shares high identity (99.8 and 98.7%, respectively) with these plasmids. Interestingly, these plasmids were isolated from outbreaks in 2008 and 2016 in different regions of the Republic of Korea and share the same plasmid backbone and genetic environment as *bla*_CTX-M-15_.

We observed this transposable unit (*bla*_CTX-M-15_-*orf477*) to be present among the same or different bacterial species. These results suggest that the plasmid genetic structure may be used as a successful genetic vehicle for dissemination among the same bacterial species or even other species.

Similar cases have been reported to be associated with co-infection of ESBL-producing pathogenic *E. coli* in other countries, such as Bangladesh and China (Ahmed et al., 2014; Xu and He, 2019). These findings indicate that co-infecting pathogenic *E. coli* strains and *Enterobacter cloacae* or uropathogenic *E. coli* isolated from urinary tract infection (UTI) patients also produce ESBL genes.

To the best of our knowledge, this is the first known report of co-infection by two types of *bla*_CTX-M_-producing pathogenic *E. coli* from clinical isolates in the Republic of Korea. Our findings suggest that the primary transmission route of resistance genes in human isolates can occur through concurrent combinations of chromosomes and plasmids. The prevalence of *bla*_CTX-M_-carrying isolates might represent a serious problem for public health and may lead to compromised efficacy of widely used broad-spectrum cephalosporins for the treatment. To prevent the spread of antimicrobial resistance genes *via* chromosome and plasmid transmission, monitoring antimicrobial susceptibilities for pathogenic *E. coli* strains and maintaining measures such as standard precautions for contaminant sources are needed.

## Data Availability Statement

The datasets presented in this study can be found in online repositories. The names of the repository/repositories and accession number(s) can be found in the article/Supplementary Material.

## Ethics Statement

This study uses strains obtained from local public health departments in the Republic of Korea. The Ethics committee of the first affiliated Korea Centers for Disease Control and Prevention decided that Institutional Review Board approval was not required, because patient information was collected anonymously and confidential patient information was not included.

## Author Contributions

JP and JuK conceived of the study, and participated in its design and draft the manuscript. JiK, Y-HJ and NP collected samples and identified isolates. JP, ES, AP, SK, and HJ carried out the experiments and analyzed the data. J-hC, KH, and KL contributed to experiment conception. All authors contributed to the article and approved the submitted version.

### Conflict of Interest

The authors declare that the research was conducted in the absence of any commercial or financial relationships that could be construed as a potential conflict of interest.

## Footnotes

^1^http://mlst.warwick.ac.uk/mlst/dbs/Ecoli

^2^https://pulsenetinternational.org/

^3^http://www.genomicepidemiology.org/

## Supplementary Material

Supplementary Table 1 Information for the pathogenic *E. coli* isolates used in this study.
