# Supplementary material for: Co-infection With Chromosomally-Located blaCTX-M-14 and Plasmid-Encoding blaCTX-M-15 in Pathogenic Escherichia coli in the Republic of Korea
Source: Front Microbiol. 2020 Nov 11;11:545591. doi: 10.3389/fmicb.2020.545591 (PMC7686028; doi:10.3389/fmicb.2020.545591)
Supplement: Supplementary Table 1 — Information for the pathogenic E. coli isolates used in this study. [file Table_1.docx]

**Supplementary Table S1.** Information of the DEC isolates in this study.

| **Isolate ID** | **Co-infection isolates** | **Collection date** | **Collection Region** | **Host disease** | **Serotype** | **Sequence type** | **Bioproject ID** | **SRA accession** |
| --- | --- | --- | --- | --- | --- | --- | --- | --- |
| 17-2481 | O | 2017 | Seoul | Diarrhoea | EAEC O44 | 414 | PRJNA595397 | SRR11047675 |
| 17-2499 | O | 2017 | Seoul | Diarrhoea | EAEC O44 | 414 | PRJNA595397 | SRR11047676 |
| 17-2502 |  | 2017 | Seoul | Diarrhoea | EAEC O44 | 414 | PRJNA595397 | SRR11047677 |
| 17-2603 |  | 2017 | Gyeonggi | Diarrhoea | EAEC O44 | 414 | PRJNA595397 | SRR11047678 |
| 17-2610 | O | 2017 | Gyeonggi | Diarrhoea | EAEC O44 | 414 | PRJNA595397 | SRR11047679 |
| 17-2645 | O | 2017 | Gyeonggi | Diarrhoea | EAEC O44 | 414 | PRJNA595397 | SRR11047682 |
| 17-2464 |  | 2017 | Seoul | Diarrhoea | ETEC O25 | 1491 | PRJNA595397 | SRR11047683 |
| 17-2480 | O | 2017 | Seoul | Diarrhoea | ETEC O25 | 1491 | PRJNA595397 | SRR11047680 |
| 17-2500 | O | 2017 | Seoul | Diarrhoea | ETEC O25 | 1491 | PRJNA595397 | SRR11047681 |
| 17-2611 | O | 2017 | Gyeonggi | Diarrhoea | ETEC O25 | 1491 | PRJNA595397 | SRR11047684 |
| 17-2624 |  | 2017 | Gyeonggi | Diarrhoea | ETEC O25 | 1491 | PRJNA595397 | SRR11047685 |
| 17-2646 | O | 2017 | Gyeonggi | Diarrhoea | ETEC O25 | 1491 | PRJNA595397 | SRR11047674 |
